# Dietary supplementation with long-chain monounsaturated fatty acids attenuates obesity-related metabolic dysfunction and increases expression of PPAR gamma in adipose tissue in type 2 diabetic KK-A^y^ mice

**DOI:** 10.1186/1743-7075-10-16

**Published:** 2013-01-30

**Authors:** Zhi-Hong Yang, Hiroko Miyahara, Yusuke Iwasaki, Jiro Takeo, Masashi Katayama

**Affiliations:** 1 Central Research Laboratory, Tokyo Innovation Center, Nippon Suisan Kaisha, Ltd., 32-3 Nanakuni 1 Chome Hachioji, Tokyo, 192-0991, Japan

**Keywords:** Long-chain monounsaturated fatty acids, Type 2 diabetes mellitus, Pparg, Adipose mass

## Abstract

The objective of present study was to examine the effect of long-chain monounsaturated fatty acids (LC-MUFAs) with chain lengths longer than 18 (i.e., C20:1 and C22:1 isomers combined) on obesity-related metabolic dysfunction and its molecular mechanisms. Type-2 diabetic KK-A^y^ mice (n = 20) were randomly assigned to the 7% soybean oil-diet group (control group) and 4% LC-MUFA concentrate-supplemented-diet group (LC-MUFA group). At 8 weeks on the diet, the results showed that plasma, liver and adipose tissue levels of C20:1 and C22:1 isomers increased significantly with LC-MUFA treatment. Supplementation with LC-MUFAs markedly reduced white fat pad weight as well as adipocyte size in the mice. The levels of plasma free fatty acids, insulin, and leptin concentration in the obese diabetic mice of the LC-MUFA group were also decreased as compared with the mice in the soybean oil-diet control group. Dietary LC-MUFAs significantly increased the mRNA expression of peroxisome proliferator-activated receptor gamma (*Pparg*), lipoprotein lipase (*Lpl*), fatty acid transport protein (*Fatp*), fatty acid translocase/CD36 (*Cd36*), as well as mRNA expression of genes involved in lipid oxidation such as carnitine palmitoyltransferase-1A (*Cpt1a*) and citrate synthase (*Cs*), and decreased the mRNA expression of inflammatory marker serum amyloid A 3 (*Saa3*) in the adipose tissues of diabetic mice. The results suggest that LC-MUFAs may ameliorate obesity-related metabolic dysfunction partly through increased expression of *Pparg* as well as its target genes, and decreased inflammatory marker expression in white adipose tissue.

## Background

Type 2 diabetes mellitus (DM) is a major global health problem and one of the major causes of morbidity and mortality. The condition is increasing in epidemic proportions in both developed and developing nations; it is estimated that there were 11 million diabetic patients in the United States in the year 2000, and the number is expected to increase to 29 million by 2050 [1-3]. The pathogenesis of type 2 DM is complex, involving progressive development of insulin resistance in peripheral tissues accompanied by defective insulin secretion from pancreatic beta cells leading to overt hyperglycemia [4]. The development of type 2 DM is caused by a combination of lifestyle and genetic factors, and the rising levels of obesity and fat accumulation as a result of a positive calorie balance is thought to be in part related to the epidemiology of type 2 DM [1]. Numerous lines of evidence support the involvement of fatty acids in type 2 DM, and many studies have demonstrated that fatty acids with different degrees of saturation have different effects on insulin sensitivity and glucose/lipid metabolism [5,6]. Compared with saturated fatty acids, increasing evidence has indicated favorable effects of monounsaturated fatty acids (MUFAs) on both obesity and DM [7-10]. On the other hand, the MUFA used in these studies was almost always oleic acid or palmitoleic acid. As such, it is unclear whether MUFAs of greater chain length (> C18) have beneficial effects on obesity-related DM. Study of Greenland Eskimos shows their high consumption of food rich in n-3 polyunsaturated fatty acids (n-3 PUFAs) as well as long-chain MUFAs (LC-MUFAs; i.e., C20:1 and C22:1), suggesting a possible correlation between LC-MUFA intake and reduced risk of obesity-related disease [11,12]. Consistently high levels of LC-MUFAs are found in the lipids of some pelagic surface fish species, such as saury [13], capelin [14], sprats [15], and herring [16], whose lipids originate from their food source, which includes zooplankton [17,18]. We previously reported that ingestion of LC-MUFA-rich fish oils such as saury oil [19] and pollock oil [20] attenuates metabolic syndrome risk factors by decreasing plasma glucose and lipid levels in diet-induced obese mice. On the other hand, besides LC-MUFAs, saury and pollock oils are also rich in n-3 PUFAs such as eicosapentaenoic acid (EPA) and docosahexaenoic acid (DHA), and therefore, it is not clear whether these protective effects were directly attributable to LC-MUFAs. To investigate the effects of LC-MUFAs on metabolic syndrome, we further produced a LC-MUFA concentrate derived from saury oil, and found that the LC-MUFA–supplemented diet also improved symptoms of metabolic syndrome in diet-induced obese mice [21]. However, it remains unknown whether dietary LC-MUFAs ameliorate obesity-related metabolic dysfunctions in experimental animals with type 2 DM.

KK-A^y^ mice, also known as Yellow KK obese mice, carry both lethal yellow obese (A^y^) and diabetic genes, and they show severe obesity, hypertriglyceridemia, hyperglycemia, hyperinsulinemia, and glucose intolerance by 8 weeks of age [22,23]. Therefore, they are widely used as a good experimental model for obesity and type 2 DM. In the current study, we examined the effect of LC-MUFA intake on diabetic risk factors in KK-A^y^ mice and further investigated the molecular mechanisms underlying these effects.

## Methods

### Animals and diets

All animal experiments were conducted in complete compliance with the National Institutes of Health’s “Guide for the Care and Use of Laboratory Animals” and were approved by the Institutional Animal Care and Use Committee at Nihon Bioresearch Inc. (Gifu, Japan), where the animals were housed for the entire experimental period. Five-week-old spontaneously diabetic male KK-A^y^ mice were obtained from CLEA Japan Inc. (Shizuoka, Japan). Mice were housed one per cage at 23 ± 1°C with a 12-h light/dark cycle and provided free access to water and standard mouse chow CRF-1 (Oriental Yeast Co., Ltd., Tokyo, Japan) for an acclimatization period of 1 week.

The fatty acid composition of the dietary oils is shown in Table 1. LC-MUFAs derived from saury oil were obtained from Nippon Suisan Kaisha, Ltd. (Tokyo, Japan); the preparation contained 58% LC-MUFAs (20:1 and 22:1 isomers combined), and the content of EPA (C20:5 n-3) and DHA (C22:6 n-3) combined was below 1%. Following the acclimatization period, the KK-A^y^ mice were randomly assigned to two groups for an 8-week feeding experiment. The control group (n = 10) was fed an AIN-93G growth diet (Oriental Yeast Co., Ltd.) containing 7% soybean oil, and the LC-MUFA group (n = 10) was fed an LC-MUFA–supplemented diet (3% soybean oil plus 4% LC-MUFA concentrate). The compositions of the experimental diets are shown in Table 2. All diet feeds were stored at −20°C and were provided fresh daily to the mice. Body weight and food intake were monitored throughout the study.

**Table 1:** Major fatty acid composition (%) of dietary oils

**Fatty acid**	**Soybean oil**	**LC-MUFA concentrate**
C14:0	0.06	3.5
C16:0	9.5	12.8
C16:1	0.1	1.3
C18:0	3.9	3
C18:1	22.8	4.8
C18:2 n-6	55.1	0.8
C18:3 n-3	7.6	0.2
C20:1 n-11	ND	16.8
C20:1 n-9	ND	4.7
C22:1 n-11	ND	34.8
C22:1 n-9	ND	1.5
C20:5 n-3	ND	0.2
C22:5 n-3	ND	0.2
C22:6 n-3	ND	0.5

ND: Not detected.

**Table 2:** Diet composition (g/100 g diet)

**Ingredient**	**Soybean oil diet (control)**	**LC-MUFA diet**
Casein	20	20
L-Cysteine	0.3	0.3
Corn starch	49.9	49.9
Sucrose	10	10
Cellulose	5	5
Mineral mixture	3.5	3.5
Vitamin mixture	1	1
Choline bitartrate	0.3	0.3
Soybean oil	7	3
LC-MUFA concentrate	—	4

At the end of the intervention period at week 8, mice were anesthetized with 4% sodium pentobarbital (Dainippon Sumitomo Pharma Co., Ltd., Osaka, Japan) in the early light phase of the light–dark cycle (fed condition). Blood was taken from the abdominal aorta, and plasma was obtained by centrifugation at 3000 rpm for 15 min and stored at −80°C until further analysis. White adipose tissue (WAT) including epididymal, mesenteric, and subcutaneous WAT was collected and weighed, and the body fat content [(epididymal WAT + mesenteric WAT + subcutaneous WAT)/body weight] was calculated. An aliquot of liver, mesenteric and subcutaneous WAT segments was snap-frozen in liquid nitrogen and stored at −80°C until analysis for either gene expression or lipid profile, and another aliquot of mesenteric WAT segments was fixed overnight at 4°C with a 4% paraformaldehyde solution in phosphate-buffered saline for further morphometric analysis.

### Biochemical analysis of plasma

Plasma concentrations of total cholesterol, triglycerides, free fatty acids (FFAs), and glucose were measured using a Cholesterol E-Test, a Triglyceride E-Test, an NEFA C-Test, and a Glucose CII-Test, respectively (Wako Pure Chemical Industries, Ltd., Osaka, Japan). Plasma levels of insulin and leptin were determined using an Insulin ELISA kit and a Leptin ELISA kit, respectively (Morinaga Institute of Biological Science, Inc., Kanagawa, Japan).

### Determination of hepatic lipid levels

Hepatic lipids were extracted from the liver according to Folch et al. [24]. The dried lipid residues were dissolved in 2-propanol containing 10% (w/w) Triton X-100 for the triglycerides and cholesterol assays. The hepatic triglycerides and cholesterol contents were measured with a triglyceride kit (Triglyceride E-Test; Wako) and a cholesterol kit (Cholesterol E-Test; Wako), respectively.

### Morphometric analysis of adipocytes

Excised mesenteric WAT was stored in 4% paraformaldehyde and then embedded in paraffin. WAT was cut into 10-μm sections that were fixed and stained with hematoxylin and eosin. The sections were viewed at 20×magnification, and images were captured with an Olympus QImaging micropublisher camera. Total adipocyte number was counted manually in five random fields for three different mice per group. Adipocyte size was calculated based on the total adipocyte number per square millimeter of adipose tissue area, and the result is expressed as the mean of adipocyte size within observed fields.

### RNA isolation and quantitative real-time reverse transcription polymerase chain reaction (QPCR)

RNA samples isolated from mesenteric or subcutaneous WAT using TRIzol reagent (Qiagen K.K., Tokyo, Japan) were reverse-transcribed to cDNA using a PrimeScript II 1^st^ Strand cDNA Synthesis kit (Takara Bio Inc., Otsu, Japan). The resulting cDNA pool was used for QPCR amplification and specific sequence detection on an Applied Biosystems 7500 Real-Time PCR System (Life Technologies Japan Ltd., Tokyo, Japan). The primers sequences are shown in Table 3. Gene expression was scaled to the expression of the housekeeping gene encoding 18S ribosomal RNA.

**Table 3:** Primers used in QPCR

**Gene**		**Primer sequence**
*Cd36*	Sense	5^′^-ATGGGCTGTGATCGGAACTG-3^′^
	Anti-sense	5^′^-AGCCAGGACTGCACCAATAAC-3^′^
*Cpt1a*	Sense	5^′^-CTCCGCCTGAGCCATGAAG-3^′^
	Anti-sense	5^′^-CACCAGTGATGATGCCATTCT-3^′^
*Cs*	Sense	5^′^-GGACAATTTTCCAACCAATCTGC-3^′^
	Anti-sense	5^′^-TCGGTTCATTCCCTCTGCATA-3^′^
*Fatp*	Sense	5^′^-CTGGGACTTCCGTGGACCT-3^′^
	Anti-sense	5^′^-TCTTGCAGACGATACGCAGAA-3^′^
*Lpl*	Sense	5^′^-TTGCCCTAAGGACCCCTGAA-3^′^
	Anti-sense	5^′^-ACAGAGTCTGCTAATCCAGGAAT-3^′^
*Pparg*	Sense	5^′^-CTCAATGCCTGATGTTTCTCCT-3^′^
	Anti-sense	5^′^-TCCAACCCTATCCCTAAAGCAA-3^′^
*Saa3*	Sense	5^′^-TGCCATCATTCTTTGCATCTTGA-3^′^
	Anti-sense	5^′^-CCGTGAACTTCTGAACAGCCT-3^′^

*Cd36*: fatty acid translocase (FAT)/CD36; *Cpt1a*: carnitine palmitoyltransferase-1a; *Cs*: citrate synthase; *Fatp*: fatty acid transport protein; *Lpl*: lipoprotein lipase; *Pparg*: peroxisome proliferator-activated receptor gamma; *Saa3*: serum amyloid A 3.

### Plasma and WAT fatty acid composition analysis

Lipids were extracted from plasma and mesenteric WAT samples using the method as described [25]. In brief, lipids were extracted by homogenizing the tissue samples in a 4:1 (v/v) methanol/hexane solution supplemented with butylated hydroxytoluene as an antioxidant. Fatty acid methyl esters were obtained by transmethylation of the lipids with acetyl chloride and heating at 80°C for 1 h under a nitrogen atmosphere. Gas chromatographic analysis of fatty acid methyl esters was performed on an Agilent 6890 N Network Gas Chromatograph System (Agilent Technologies Japan, Ltd., Tokyo, Japan). Fatty acids were identified by comparison of retention times with those of purified standards including Nu-Chek Prep 462 (Elysian, MN, USA) and polyunsaturated fatty acid (PUFA) No. 1, Marine Source (Sigma-Aldrich, St. Louis, MO, USA).

### Statistical analysis

Results are expressed as mean ± SE. Student's *t*-test was used for the statistical analysis. Values were considered to be significantly different when the *p* value was less than 0.05.

## Results

### Effect of LC-MUFAs on body and organ weights and body fat content

As shown in Table 4, there were no significant differences in food intake and body weight between the control and the LC-MUFA groups. However, dietary treatment with LC-MUFAs for 8 weeks decreased epididymal WAT, mesenteric WAT, subcutaneous WAT, and the body fat content of mice by 10.9% (*p* < 0.05), 8.9% (*p* = 0.1), 31.2% (*p* < 0.01), and 16.2% (*p* < 0.01), respectively. Despite the difference in liver weights (control: 4.0 ± 0.1 g/100 g BW vs. LC-MUFA diet: 4.8 ± 0.2 g/100 g BW; *p* < 0.05), there were no differences between diet groups in hepatic triglyceride and total cholesterol contents.

**Table 4:** **Effect of LC-MUFAs on body weight, body fat contents and plasma metabolites in KK-A**^**y **^**mice for 8 wk**

	**Soybean oil diet (Control)**	**LC-MUFA diet**
Food intake (g/day)	4.4 ± 0.3	4.4 ± 0.2
Initial body mass (g)	29.9 ± 0.4	29.5 ± 0.5
Final body mass (g)	42.4 ± 0.8	40.1 ± 1.1
Epididymal WAT (g/100 g BW)	4.6 ± 0.2	4.1 ± 0.2*
Mesenteric WAT (g/100 g BW)	2.7 ± 0.1	2.4 ± 0.1
Subcutaneous WAT(g/100 g BW)	3.2 ± 0.3	2.2 ± 0.2**
Body fat contents (g/100 g BW)	10.5 ± 0.4	8.8 ± 0.3**
Liver (g/100 g BW)	4.0 ± 0.1	4.8 ± 0.2*
Hepatic triglyceride (mg/g Liver)	60.5 ± 4.8	68.7 ± 2.7
Hepatic total cholesterol (mg/g Liver)	4.0 ± 0.6	3.8 ± 0.4
Plasma total cholesterol (mg/dL)	180.4 ± 7.3	156.4 ± 8.2*
Plasma triglycerides (mg/dL)	204.4 ± 13.5	220.4 ± 21.3
Plasma FFAs (μEq/mL)	696.4 ± 25	553.8 ± 30.7**
Plasma glucose (mg/dL)	486.4 ± 30.1	475.6 ± 42.3
Plasma insulin (ng/mL)	82.8 ± 8.9	61 ± 4.1*
Plasma leptin (ng/mL)	19.4 ±0.7	16.3 ±0.7*

Each value represents the mean ± SE (n = 10). BW: body weight; FFAs: free fatty acids; WAT: white adipose tissue; Body fat contents: (epididymal adipose tissue + mesenteric adipose tissue + subcutaneous adipose tissue)/body weight. * *p* < 0.05; ** *p* < 0.01 compared with the control group.

### Effect of LC-MUFAs on plasma metabolites and hormones

The effects of dietary LC-MUFA treatment on circulating concentrations of total cholesterol, triglycerides, FFAs, glucose, insulin, and leptin are shown in Table 4. LC-MUFAs significantly decreased plasma levels of total cholesterol by 13.3% (*p* < 0.05), FFAs by 20.5% (*p* < 0.01), insulin by 26.3% (*p* < 0.05), and leptin by 15.9 (*p* < 0.05), although plasma levels of triglycerides and glucose did not significantly differ between the LC-MUFA and control groups.

### Effect of LC-MUFAs on adipocyte size

**Figure 1:**
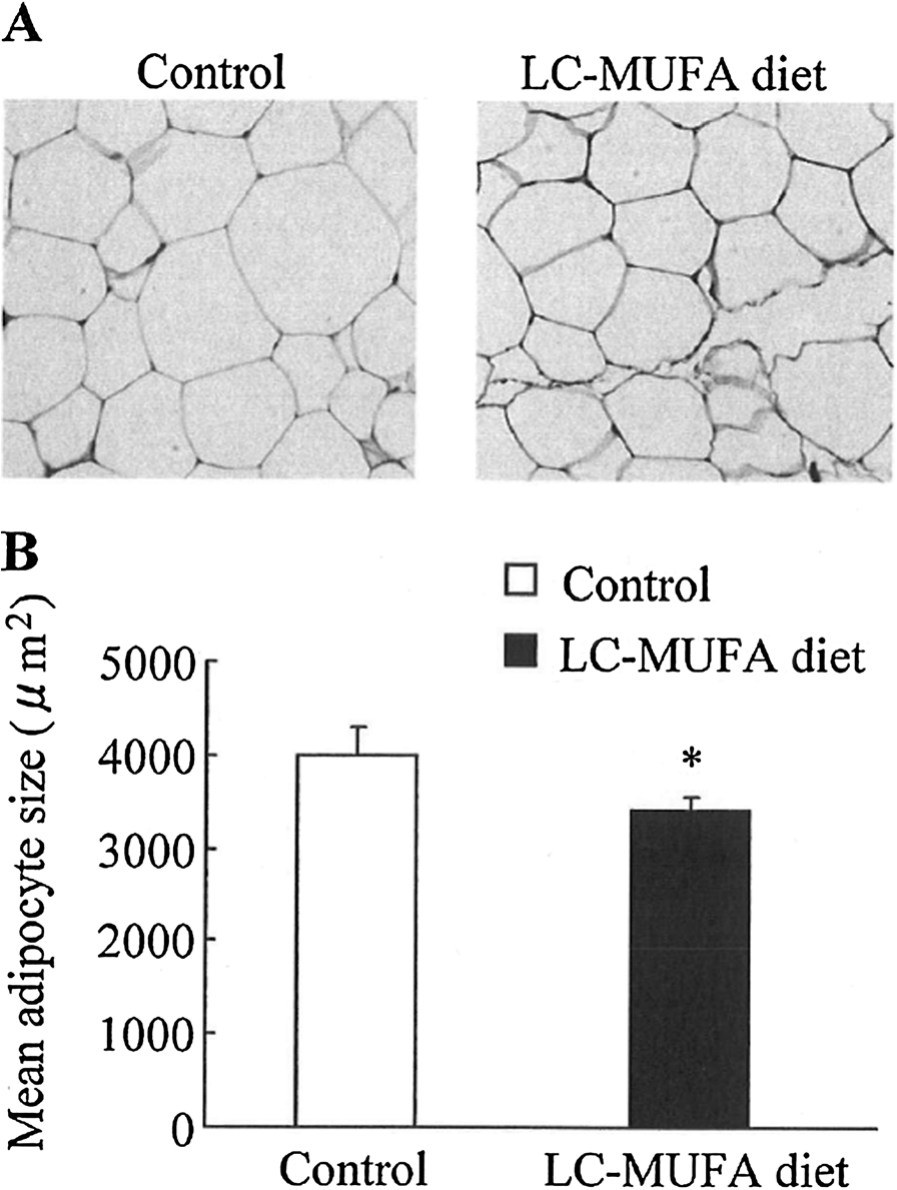
**Adipocyte size in KK-A**^**y **^**mice fed the control diet or LC-MUFA diet for 8 weeks.** Mesenteric WAT sections were stained with hematoxylin and eosin (**A**), and adipocyte diameters were measured (**B**). The diameters of adipocytes are presented as mean values for a 1-mm^2^ area of WAT section. Values represent the mean ± SE, n = 5. *p < 0.05 compared with the control group.

Compared with the control, adipocyte size in the LC-MUFA group notably decreased (*p* < 0.05; Figure 1). Adipocytes from the LC-MUFA group were ~16% smaller than adipocytes from the control group.

### Effect of LC-MUFAs on mRNA expression of peroxisome proliferator-activated receptor gamma (Pparg) target genes in adipose tissues

**Figure 2:**
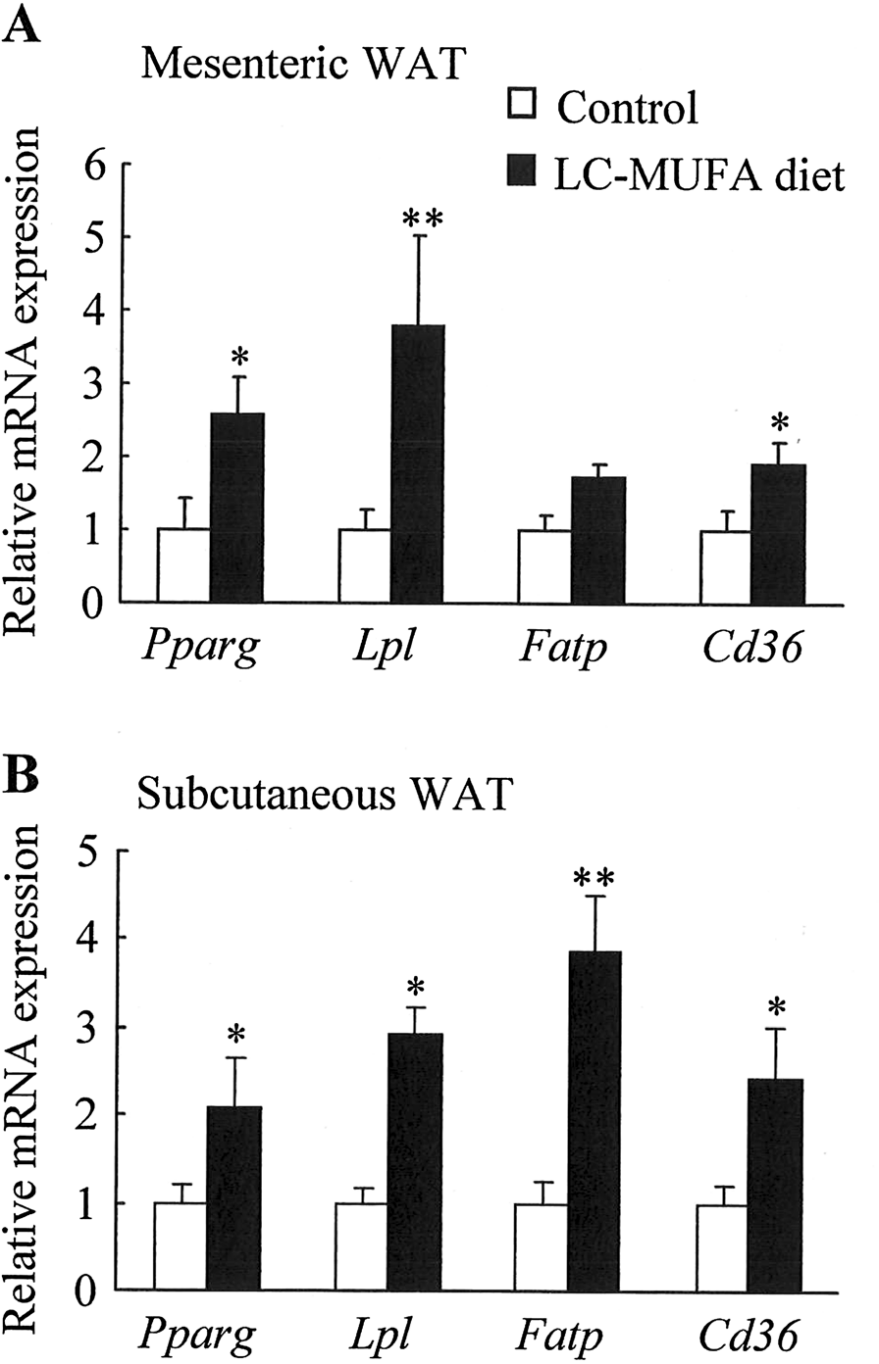
**mRNA expression of Pparg target genes in adipose tissue of KK-A**^**y **^**mice fed the control diet or LC-MUFA diet for 8 weeks.** Expression of the gene encoding peroxisome proliferator-activated receptor gamma (*Pparg*) and its target genes lipoprotein lipase (*Lpl*), fatty acid transport protein (*Fatp*), and fatty acid translocase/CD36 (*Cd36*) in adipose tissues was quantified by QPCR analysis. (**A**) Mesenteric WAT, (**B**) subcutaneous WAT. Values represent the mean ± SE, n = 10. *p < 0.05, **p < 0.01 compared with the control group.

To explore the potential molecular mechanism of reducing fat pad weight and obesity-related metabolic dysfunction by LC-MUFAs, the expression of the gene encoding *Pparg* and its target genes in mesenteric and subcutaneous WAT was determined by QPCR. The LC-MUFA–supplemented diet upregulated the expression of *Pparg*, *Lpl* (lipoprotein lipase), and *Cd36* (fatty acid translocase/CD36) mRNA by 160% (*p* < 0.05), 280% (*p* < 0.01), and 92% (*p* < 0.05), respectively, in mesenteric WAT (Figure 2A). LC-MUFA intake also upregulated the expression of *Pparg*, *Lpl*, *Fatp* (fatty acid transport protein), and *Cd36* mRNA by 110% (*p* < 0.05), 190% (*p* < 0.05), 280% (*p* < 0.01), and 140% (*p* < 0.05), respectively, in subcutaneous WAT (Figure 2B).

### Effect of LC-MUFAs on mRNA expression of genes involved in lipid oxidation in adipose tissues

**Figure 3:**
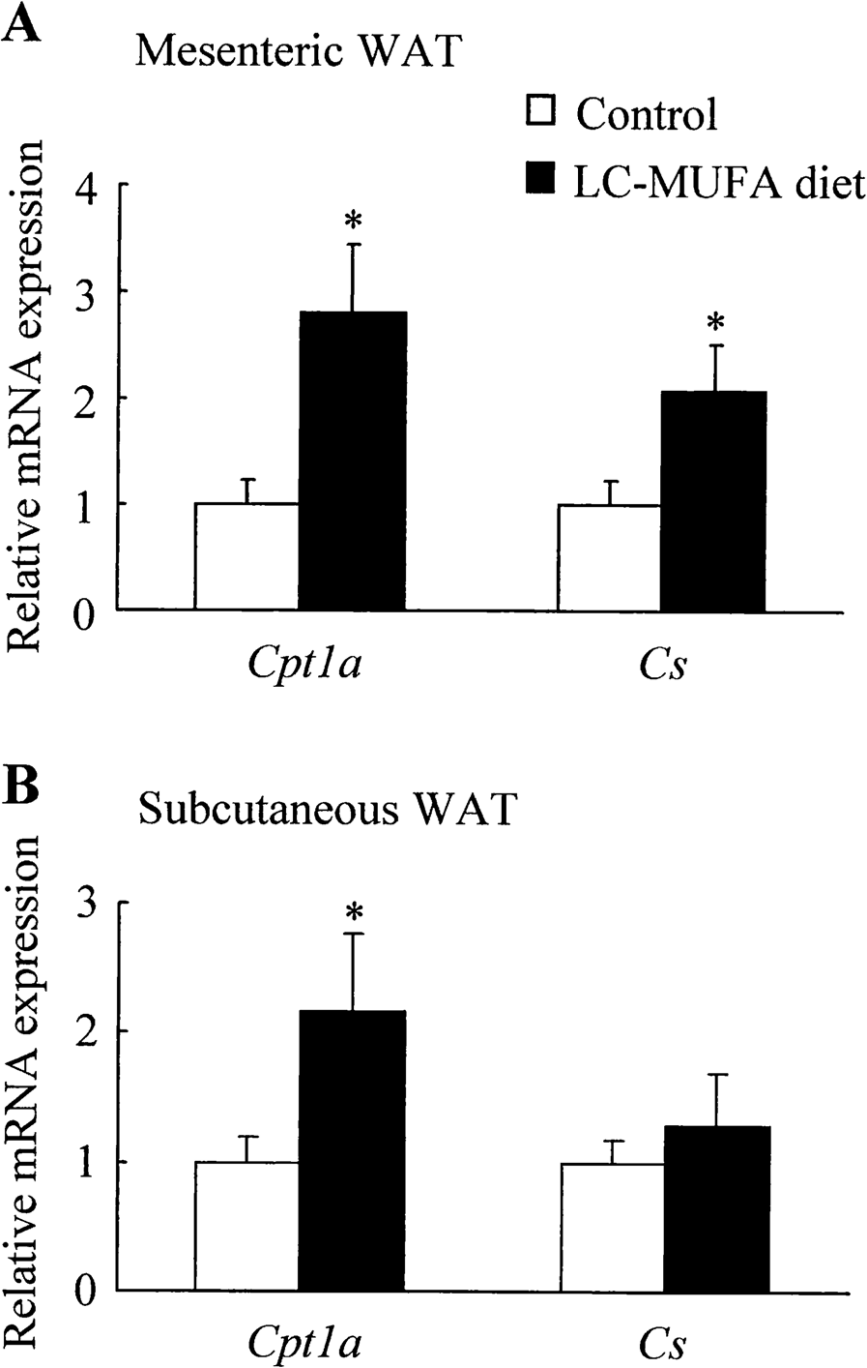
**mRNA expression of genes involved in lipid oxidation in adipose tissue of KK-A**^**y **^**mice fed the control diet or LC-MUFA diet for 8 weeks.** Expression of carnitine palmitoyltransferase 1a (*Cpt1a*) and citrate synthase (*Cs*) in adipose tissues were quantified by QPCR analysis. (**A**) Mesenteric WAT, (**B**) subcutaneous WAT. Values represent the mean ± SE, n = 10. *p < 0.05 compared with the control group.

Intake of LC-MUFAs increased expression of *Cpt1a* (carnitine palmitoyltransferase 1a) by 180% (*p* < 0.05) and *CS* (citrate synthase) by 95.4% (*p* < 0.05) in mesenteric WAT (Figure 3A). Similarly, LC-MUFA intake upregulated expression of *Cpt1a* mRNA by 120% (*p* < 0.05) in subcutaneous WAT (Figure 3B).

### Effect of LC-MUFAs on mRNA expression of inflammatory marker gene in adipose tissues

**Figure 4:**
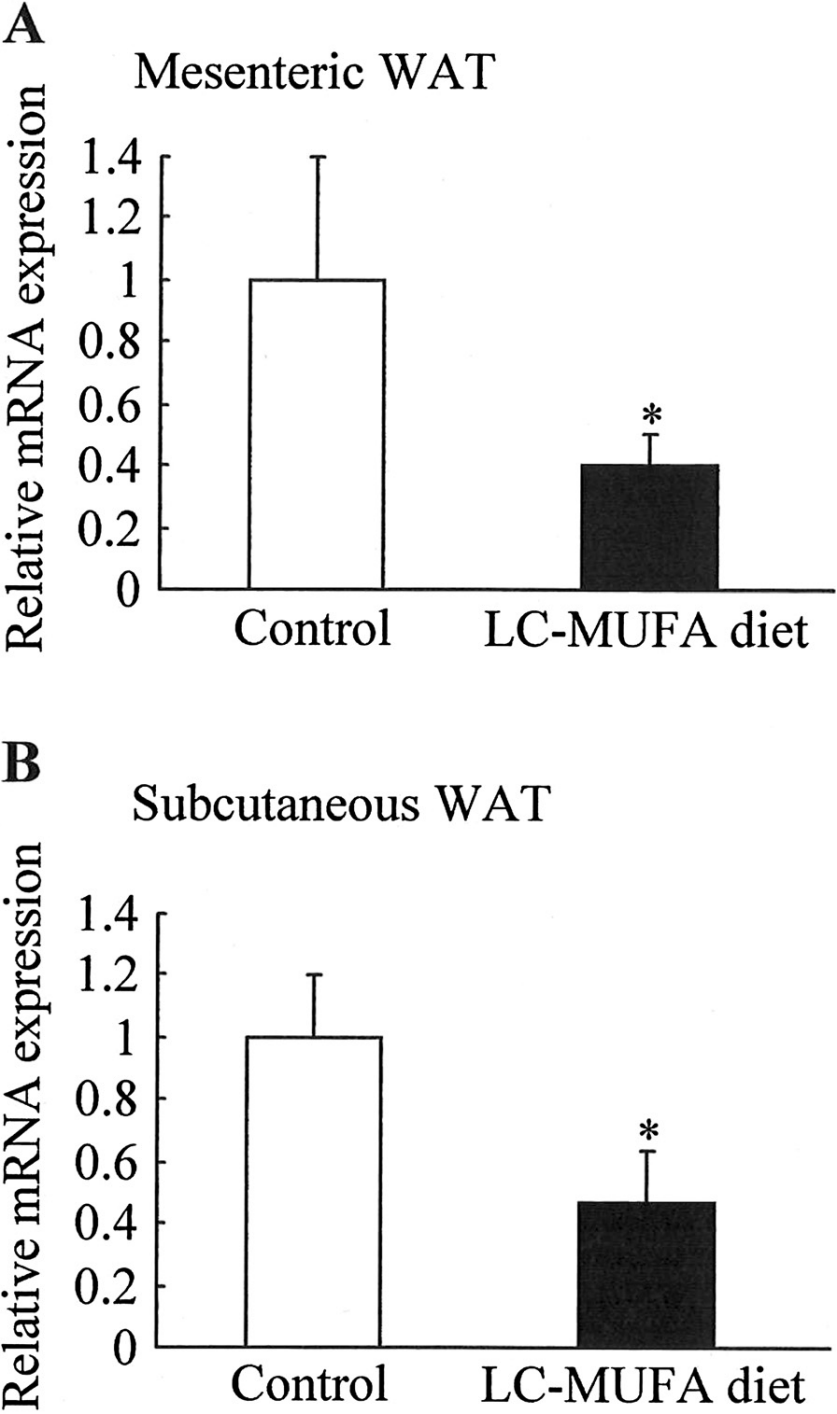
**mRNA expression of inflammatory marker gene in adipose tissues of KK-A**^**y **^**mice fed the control diet or LC-MUFA diet for 8 weeks.** Expression of serum amyloid A 3 (*Saa3*) in adipose tissues were quantified by QPCR analysis. (**A**) Mesenteric WAT, (**B**) subcutaneous WAT. Values represent the mean ± SE, n = 10. *p < 0.05 compared with the control group.

To investigate whether LC-MUFAs reduce adipose inflammation in KK-A^y^ mice, we measured mRNA expression of *Saa3* (serum amyloid A 3) in adipose tissues. As shown in Figure 4, LC-MUFA diet decreased expression of *Saa3* by 60% (*p* < 0.05) in mesenteric WAT (Figure 4A), and by 52% (*p* < 0.05) in subcutaneous WAT (Figure 4B).

### Effect of LC-MUFAs on fatty acid composition of plasma, liver, and WAT

As shown in Table 5, the percentage of LC-MUFAs (C20:1 and C22:1 combined) as well as total MUFAs significantly increased in the LC-MUFA group in plasma and liver of KK-A^y^ mice after treatment for 8 weeks. In the LC-MUFA group, the combined levels of C20:1 and C22:1 isomers in plasma and liver increased by 370% (*p* < 0.001) and 200% (*p* < 0.001), respectively. Ingestion of LC-MUFA reduced linoleic acid (C18:2 n-6) levels significantly in plasma and liver by 16% (*p* < 0.01) and 45.7% (*p* < 0.001), respectively, and accordingly decreased total n-6 PUFA levels significantly in plasma and liver by 19.5% (*p* < 0.01) and 45.4% (*p* < 0.001), respectively. Compared to the control group, the plasma levels of EPA and total n-3 PUFA levels increased significantly in the LC-MUFA diet group by 132.1% (*p* < 0.001) and 16.1% (*p* < 0.01), respectively. In contrast, LC-MUFA diet decreased DHA and total n-3 PUFA levels by 19.2% (*p* < 0.05) and 24.3% (*p* < 0.01). Nevertheless, LC-MUFA diet increased plasma and liver n-3/n-6 ratio by 42% (*p* < 0.001) and 35% (*p* < 0.001), respectively.

**Table 5:** **Effect of LC-MUFAs on fatty acid composition (%) in plasma and liver in KK-A**^**y **^**mice**

**Fatty acid**	**Plasma**		**Liver**	
	**Soybean oil diet (control)**	**LC-MUFA diet**	**Soybean oil diet (control)**	**LC-MUFA diet**
14:0	0.19 ± 0.04	0.30 ± 0.02*	0.38 ± 0.06	0.56 ± 0.02*
16:0	21.68 ± 0.59	20.34 ± 0.49	22.11 ± 0.32	23.62 ± 0.60*
18:0	12.05 ± 0.69	9.31 ± 0.41**	6.12 ± 0.34	4.36 ± 0.24***
20:0	0.35 ± 0.04	0.35 ± 0.01	0.41 ± 0.02	0.39 ± 0.02
SFA	34.26 ± 0.96	30.31 ± 0.73**	29.02 ± 0.38	28.94 ± 0.61
16:1 n-7	1.57 ± 0.26	2.61 ± 0.22**	3.51 ± 0.20	5.14 ± 0.39**
18:1 n-9	12.21 ± 1.62	17.84 ± 0.79**	28.74 ± 1.11	36.23 ± 1.01***
18:1 n-7	2.41 ± 0.19	2.99 ± 0.13*	3.68 ± 0.19	4.67 ± 0.22**
20:1 n-11	ND	0.73 ± 0.06***	ND	1.27 ± 0.07***
20:1 n-9	0.41 ± 0.05	0.74 ± 0.02***	0.78 ± 0.03	0.91 ± 0.04*
22:1 n-11	ND	0.48 ± 0.09**	ND	0.36 ± 0.03***
22:1 n-9	0.01 ± 0.01	0.03 ± 0.01	0.12 ± 0.01	0.17 ± 0.01**
LC-MUFA	0.42 ± 0.06	1.97 ± 0.18***	0.90 ± 0.03	2.70 ± 0.13***
MUFA	16.61 ± 2.06	25.39 ± 0.92**	36.83 ± 1.37	48.74 ± 0.98***
18:2 n-6	26.71 ± 1.11	22.37 ± 0.47**	18.43 ± 0.73	10.01 ± 0.46***
18:3 n-6	0.27 ± 0.03	0.32 ± 0.01	0.20 ± 0.01	0.13 ± 0.01***
20:2 n-6	0.32 ± 0.04	0.26 ± 0.01	0.25 ± 0.03	0.12 ± 0.01***
20:3 n-6	1.35 ± 0.09	1.52 ± 0.07	0.62 ± 0.03	0.48 ± 0.03**
20:4 n-6	9.98 ± 1.17	6.61 ± 0.44***	5.11 ± 2.71	2.71 ± 0.24***
n-6 PUFA	38.62 ± 1.71	31.08 ± 0.56**	24.61 ± 0.97	13.44 ± 0.57***
18:3 n-3	0.89 ± 0.06	0.53 ± 0.05***	1.10 ± 0.04	0.41 ± 0.03***
20:5 n-3	0.62 ± 0.07	1.39 ± 0.04***	0.32 ± 0.03	0.39 ± 0.03
22:5 n-3	0.42 ± 0.06	0.54 ± 0.02	0.10 ± 0.01	0.19 ± 0.02**
22:6 n-3	5.42 ± 0.37	6.08 ± 0.32	3.33 ± 0.20	2.69 ± 0.20*
n-3 PUFA	7.32 ± 0.28	8.54 ± 0.27**	4.85 ± 0.25	3.67 ± 0.22**
n-3/n-6	0.19 ± 0.01	0.27 ± 0.01***	0.20 ± 0.01	0.27 ± 0.01***

Each value represents the mean ± SE (n = 10). ND: Not detected; WAT: white adipose tissue; SFA: saturated fatty acids; MUFA: monounsaturated fatty acids; LC-MUFA: long-chain monounsaturated fatty acids (C20:1 and C22:1 isomers combined); PUFA: polyunsaturated fatty acids; * *p* < 0.05; ** *p* < 0.01; *** *p* < 0.001 compared with the control group.

Table 6 shows fatty acid compositions in adipose tissues including mesenteric WAT and subcutaneous WAT with soybean oil diet and LC-MUFA diet. LC-MUFA diet increased LC-MUFAs in mesenteric WAT by 740% (*p* < 0.001), and those in subcutaneous WAT by 883% (*p* < 0.001). In contrast, LC-MUFA diet decreased total n-6 and n-3 PUFA levels in both mesenteric WAT and subcutaneous WAT. In the LC-MUFA group, total n-6 PUFA levels in mesenteric WAT and subcutaneous WAT decreased by 45.8% (*p* < 0.001) and 42% (*p* < 0.001), respectively. With LC-MUFA diet, total n-3 PUFA levels in mesenteric WAT and subcutaneous WAT decreased by 48.1% (*p* < 0.001) and 49.3% (*p* < 0.001), respectively.

**Table 6:** **Effect of LC-MUFAs on fatty acid composition (%) in mesenteric and subcutaneous WAT in KK-A**^**y **^**mice**

**Fatty acid**	**Mesenteric WAT**		**Subcutaneous WAT**	
	**Soybean oil diet (control)**	**LC-MUFA diet**	**Soybean oil diet (control)**	**LC-MUFA diet**
14:0	0.81 ± 0.02	1.18 ± 0.02*	0.76 ± 0.09	1.31 ± 0.15* *
16:0	18.92 ± 0.27	20.97 ± 0.41**	15.42 ± 0.22	16.29 ± 0.98
18:0	2.31 ± 0.21	2.26 ± 0.09	1.45 ± 0.38	1.18 ± 0.42
20:0	0.10 ± 0	0.11 ± 0.01	0.08 ± 0	0.12 ± 0.01
SFA	22.13 ± 0.37	24.53 ± 0.43**	17.72 ± 0.31	18.90 ± 0.43
16:1 n-7	4.42 ± 0.21	5.24 ± 0.41	6.27 ± 0.89	7.27 ± 1.54
18:1 n-9	31.19 ± 0.39	35.68 ± 0.45***	30.06 ± 0.64	35.88 ± 0.85***
18:1 n-7	2.46 ± 0.13	3.03 ± 0.05**	2.64 ± 0.20	2.99 ± 0.39*
20:1 n-11	0.08 ± 0	3.03 ± 0.11***	0.05 ± 0	3.14 ± 0.32***
20:1 n-9	0.56 ± 0.01	1.27 ± 0.04***	0.48 ± 0.04	1.29 ± 0.09***
22:1 n-11	ND	1.29 ± 0.06***	ND	1.36 ± 0.19***
22:1 n-9	0.04 ± 0	0.13 ± 0.01***	0.03 ± 0	0.14 ± 0.02***
LC-MUFA	0.68 ± 0.01	5.72 ± 0.21***	0.57 ± 0.01	5.94 ± 0.19***
MUFA	38.63 ± 0.49	49.67 ± 0.91***	39.54 ± 0.38	52.07 ± 0.71***
18:2 n-6	33.58 ± 0.52	20.14 ± 0.52***	33.89 ± 0.98	19.62 ± 1.69***
18:3 n-6	0.05 ± 0	0.04 ± 0.01	0.06 ± 0	0.07 ± 0.01*
20:2 n-6	0.13 ± 0	0.09 ± 0.01***	0.11 ± 0.01	0.08 ± 0.01***
20:3 n-6	0.05 ± 0	0.04 ± 0*	0.06 ± 0	0.04 ± 0.01**
20:4 n-6	0.11 ± 0.01	0.09 ± 0.01	0.13 ± 0.01	0.09 ± 0.02***
n-6 PUFA	33.92 ± 0.52	18.37 ± 0.71***	34.26 ± 0.31	19.91 ± 0.63***
18:3 n-3	2.09 ± 0.22	1.09 ± 0.05**	2.64 ± 0.14	1.19 ± 0.10***
20:5 n-3	0.03 ± 0	0.01 ± 0.01	0.02 ± 0.01	0.03 ± 0.01
22:5 n-3	0.01 ± 0	0.01 ± 0	0.01 ± 0.01	0.02 ± 0.01
22:6 n-3	0.04 ± 0	0.14 ± 0.01***	0.04 ± 0.01	0.14 ± 0.02***
n-3 PUFA	2.16 ± 0.22	1.12 ± 0.14***	2.72 ± 0.05	1.38 ± 0.04***
n-3/n-6	0.06 ± 0.01	0.06 ± 0	0.08 ± 0	0.07 ± 0

Each value represents the mean ± SE (n = 10). ND: Not detected; WAT: white adipose tissue; SFA: saturated fatty acids; MUFA: monounsaturated fatty acids; LC-MUFA: long-chain monounsaturated fatty acids (C20:1 and C22:1 isomers combined); PUFA: polyunsaturated fatty acids; * *p* < 0.05; ** *p* < 0.01; *** *p* < 0.001 compared with the control group.

## Discussion

It is well established that patients with type 2 DM often have a co-occurrence of insulin resistance, hyperinsulinemia, hyperglycemia, dyslipidemia, and/or obesity [26-28]. In the present study, we showed that dietary administration of LC-MUFAs for 8 weeks attenuated hyperinsulinemia and hyperlipidemia and decreased fat pad weight in diabetic KK-A^y^ mice.

Insulin resistance has been assigned a central place in the metabolic disturbances associated with obesity and type 2 DM, and compensatory hyperinsulinemia stemming from peripheral insulin resistance is a well-recognized metabolic disturbance in type 2 DM [29-31]. Furthermore, it has been demonstrated that mice transfected with extra copies of the insulin gene produce elevated insulin levels, and they show insulin resistance, hyperglycemia, and hypertriglyceridemia, suggesting that insulin itself is an important contributor to insulin resistance [32]. Besides hyperinsulinemia, obesity-derived hyperlipidemia is one of the suggested possible mechanisms for type 2 DM. Serum FFAs, which are elevated in most obese subjects, induce metabolic insulin resistance and inhibit insulin signaling through different mechanisms [33,34]. The LC-MUFA diet decreased plasma insulin and FFA concentrations that were markedly elevated in spontaneous type 2 diabetic KK-A^y^ mice, suggesting that treatment of diabetic mice with LC-MUFAs may favorably impact the risk factors for type 2 DM. On the other hand, non-fasting levels of plasma triglycerides and glucose were not significantly different between the control and LC-MUFA diet group. It is therefore suggested that the antidiabetic effect of LC-MUFAs in KK-A^y^ mice was mild. In addition, although there were no significant differences in hepatic triglycerides and total cholesterol, LC-MUFA intake increased liver weight. It is not yet clear whether this is due to the changes in the fatty acid composition of the liver with LC-MUFA administration, and further studies are needed to address whether LC-MUFAs have any impact on liver health and fatty liver in particular.

Because type 2 DM is closely associated with obesity, ~80–90% of people diagnosed with type 2 DM are also diagnosed as being overweight or obese, and several studies of human subjects have shown that the risk of developing DM is reduced by weight loss [35,36]. Research suggests that adipose tissue not only serves as a storage site for fat but also functions as an endocrine organ [37,38]. Adipose tissue grows by two mechanisms: hyperplasia and hypertrophy, the latter occurring prior to hyperplasia to meet the need for additional fat storage capacity as obesity progresses [39]. In fact, obesity is characterized by adipocyte hypertrophy followed by increased angiogenesis, immune cell infiltration, extracellular matrix overproduction, and, consequently, by increased production of proinflammatory adipocytokines and FFAs, which are potentially involved in the pathogenesis of insulin resistance. In the present study, whereas there were no differences in body weight gain between the LC-MUFA group and control group, dietary treatment of KK-A^y^ mice with LC-MUFAs reduced fat accumulation and caused decreased adipocyte size, which may in turn have improved lipid metabolism and attenuated compensatory hyperinsulinemia. The LC-MUFA diet increased plasma and organ levels of C20:1 and C22:1 isomers, suggesting a negative correlation between LC-MUFA intake and risk factors for type 2 DM in KK-A^y^ mice. Plasma, liver and WAT levels of linoleic acid (C18:2 n-6), a major fatty acid in soybean oil used in the control diet, decreased significantly in mice fed the LC-MUFA diet, however, suggesting a minor role for linoleic acid in the improvement of type 2 DM with LC-MUFA treatment. Worthy of note, the predominant LC-MUFA isomers in the current study is C22:1 n-11, an isomer of erucic acid (C22:1 n-9) which is an important fatty acid of rapeseed oil, and LC-MFUA diet increased plasma and organ levels of C22:1 n-11 significantly. Given that there is continuing controversy as to the health effects of erucic acid [40-42], further study is required to examine the physiologic similarities and differences between the C22:1 isomers. Furthermore, LC-MUFA diet increased palmitoleic acid (C16:1 n-7) levels in plasma and liver significantly, and in adipose tissues non-significantly (*p* < 0.15). Although it has been proposed some beneficial effects of palmitoleic aicd in cell culture and animal models [43,44], human plasma palmitoleic acid content has been reported to be a consistent and independent predictor in incident diabetes [45,46]. It is therefore suggested that no all MUFAs are uniformly beneficial, and we could not exclude the possibility that a rise in palmitoleic acid with LC-MUFA diet could have some potential negative implications in a diabetic animal model.

To explore the potential positive impact of reducing adipocyte size and lowering plasma FFAs, we evaluated the expression of *Pparg*, *Lpl*, *Fatp*, and *Cd36* mRNA in adipose tissue. Pparg, which is expressed predominantly in adipose tissue, plays crucial roles in regulating adipocyte differentiation, fatty acid metabolism, and insulin signal transduction [47,48]. Overexpression of *Pparg* in mature 3T3-L1 adipocytes results in a decrease in both cell size and intracellular triglyceride content, and Pparg activation results in a marked improvement of insulin and glucose parameters resulting from an improvement of whole-body insulin sensitivity in type 2 diabetic patients [49-51]. The *Pparg* target genes *Lpl*, *Fatp*, and *Cd36* are important control elements in FFA homeostasis [52,53]. FFAs liberated from circulating lipoproteins through hydrolysis by LPL bind to albumin, and cell surface receptors such as Fatp and Cd36 facilitate rapid uptake and coordinate the import of FFAs. Our data demonstrate that *Pparg* mRNA level increased in adipose tissue of KK-A^y^ mice fed LC-MUFAs, which was paralleled by increases in *Lpl*, *Fatp*, and *Cd36* mRNAs, suggesting that reduction in adipocyte size and an increase in FFA uptake in adipose tissue were partly attributed to upregulation of *Pparg* and its target genes, *Lpl*, *Fatp*, and *Cd36*. Furthermore, concomitant with increase in *Pparg* gene expression, plasma leptin levels were decreased with the LC-MUFA diet. Leptin, a 16 KDa protein hormone that plays a key role in regulating adipose tissue mass and energy balance, is secreted primarily by adipocytes [54]. Circulating leptin levels are highly correlated with body fat stores, and high plasma leptin levels are observed in obese subjects as a result of increased production in enlarged fat cells [55]. Studies have demonstrated that leptin is located at Pparg downstream, and Pparg activation inhibits leptin gene transcription [56]. It is therefore suggested that LC-MUFAs decreased plasma leptin levels may possibly through activating Pparg in adipose. Furthermore, it has been demonstrated that adipose inflammation plays key roles in the vascular complications of obesity, insulin resistance, as well as type 2 DM, and hypertrophic adipocytes within adipose tissue directly augment systemic inflammation [57]. The present study shows that LC-MUFA feeding downregulated the expression of inflammatory marker Saa3 in adipose tissue with concomitant decreases in adipocyte size. These results suggest possible mechanisms for the beneficial effects of a LC-MUFA-rich diet.

Insulin resistance in subjects with type 2 DM and obesity is connected with an imbalance between the availability and the oxidation of lipids [58]. Cpt1a and Cs are associated with fatty acid utilization and oxidation capacity [59,60], and the increase in *Cpt1a* and *Cs* expression observed in the current study was possibly related to the elevated uptake of circulating FFAs by adipose tissue. Studies have shown that inhibition of Cpt1a increases lipid deposition and exacerbates insulin resistance when animals are placed on a high-fat diet, whereas overexpression of *Cpt1a* protects myotubes against lipid-induced insulin resistance [61-63]. The present study suggests that a diet supplemented with LC-MUFAs may promote fatty acid oxidation by upregulating *Cpt1a* and *Cs* expression, which is possibly associated with a diminution of risk factors for type 2 DM.

## Conclusions

In conclusion, dietary treatment of KK-A^y^ mice with LC-MUFAs improved their diabetic condition. Fat pad weight as well as adipocyte size, hyperlipidemia, hyperinsulinemia, and hyperleptinemia were reduced in response to the LC-MUFA diet, most likely partly attributable to upregulation of *Pparg* and its target genes. An increase in LC-MUFA levels in plasma and WAT following consumption of an LC-MUFA diet is likely to be involved in the observed beneficial changes to metabolic indicators.

## Abbreviations

Cd36: Fatty acid translocase; Cpt1a: Carnitine palmitoyltransferase-1a; Cs: Citrate synthase; DM: Diabetes mellitus; Fatp: Fatty acid transport protein; FFA: Free fatty acid; LC-MUFA: Long-chain monounsaturated fatty acid; Lpl: Lipoprotein lipase; PUFA: Polyunsaturated fatty acid; Pparg: Peroxisome proliferator-activated receptor gamma; QPCR: Quantitative real-time polymerase chain reaction; Saa3: Serum amyloid A 3.

## Competing interests

The authors declare that they have no competing interests.

## Authors’ contributions

ZHY participated in the planning, analysis, and manuscript preparation. HM and YI participated in the experimental work. JT and MK participated in the planning and organization of the study. All authors read and approved the final manuscript.
